# Exemplifying inclusion through mediated intergroup contact between cis and trans people and mitigating prejudice in select Tamil films, *Peranbu* (2018) and *Natchathiram Nagargiradhu* (2022)

**DOI:** 10.3389/fsoc.2026.1690839

**Published:** 2026-03-18

**Authors:** Mary Angelene J., Alamelu C.

**Affiliations:** School of Social Sciences and Languages, Vellore Institute of Technology, Chennai, Tamil Nadu, India

**Keywords:** inclusivity, intergroup contact, media representation, prejudice reduction, transgender

## Abstract

**Introduction:**

The paper focuses on the progressive portrayal of trans women in select Tamil films namely, Ram’s *Peranbu (2018)* and Ranjith’s *Natchathiram Nagargiradhu (2022)* as there has been a significant increase in the representations of trans communities in Tamil cinema. The objective is to assess whether the select films have a valuable role to play in the prejudice reduction of trans communities.

**Methods:**

To achieve this end, the paper employs the Integrated Mediated Intergroup Contact (IMIC) model and Trans-Identity theoretical lens to undertake a qualitative textual analysis of the visual texts.

**Results:**

The resultant combined framework of IMIC model and Trans-Identity theory suggests that positive trans representations involving dignified intergroup contact have more chances of eliciting desirable attitudes towards trans people and inclusive intergroup behaviors.

**Discussion:**

The analysis spotlights that positively mediated intergroup contact between cis and trans communities through an integrated framework of both parasocial and vicarious contact can lead to empathy and self efficacy which would result in the comprehension and validation of lived experiences of trans people reducing prejudice and eliminating trans discrimination.

## 1 Introduction

Transgender communities have been ostracized by the predominant cisgender population in India due to their divergence from the rigid heteronormative structures of gender, despite being legally acknowledged as the third gender in the year 2014 (Pillai and Sreenivasulu, 2024). Tracing the history of transgender in India through the documentation of Mishra (2023), it is intriguing to note that before colonization, trans communities were accorded considerable esteem, holding influential positions within the army and other administrative circles as evident in Vedic stories, epics and scriptures. They were appointed bodyguards, counselors, royal messengers (Penrose, 2001) and had a significant role in Harems, the abode of the royalty (Varghese and Tanupriya, 2023). India had various transgender categories with the labels “hijra, jogta or jogappa, aravani or thirunangai, shiv-shakthi, kothis, khwaja sira, khunsa, zennana, kinnar, and others” that were not in the scope of Eurocentric gender binaries (Billard et al., 2020; Choudhury and Harini, 2023; Tiwari and Sharma, 2023). These social identities were erased by the British during the colonial period and categorized trans people with their assigned sex at birth (Billard et al., 2020). In addition, detrimental laws were coded to infringe on them as criminal tribes and their lives were jeopardized as per these legal codes (Mishra, 2023).

Post colonization, fluid identities were conglomerated globally into a singular Eurocentric ‘transgender’ identity label (Billard et al., 2020) and disseminated through global media and globalized industries of culture as observed by Chiang (2014), Hegarty (2017) and Leung (2016). But the propagation of transgender identity through media, as Keegan et al. (2018) assert, did not bring proper awareness or enhance their living conditions, as the representations were misleading. These projections indicate non inclusivity and gatekeeping of trans realities in narratives to sustain binaries (Keegan et al., 2018). Influenced by the Eurocentric propaganda of transgender identity label, Indian mainstream films symbolically annihilated transgender people, which refers to the “absence of representations, underrepresentation of a particular social group, or a markedly strong pattern of negative representations” (Hilton-Morrow and Battles, 2015). They either invariably ignored the inclusion of individuals belonging to the non-binary gender spectrum, or included them intending to typecast them with negative roles and disparagingly ridicule them catering to transphobic perceptions of the society (Kaur and Singh, 2025; Qadiri, 2023; Mishra, 2023). Such transphobic interactions between cis and trans communities contribute to the broader systemic neglect, harassment and injustice towards trans people as the audience relate reel to real lives, with and without discretion (Tiwari and Sharma, 2023; Tilak and Singh, 2019). Bhattacharya (2023) also affirms that marginalization of trans community has happened due to repeatedly trivializing them on screen and the lack of profound and sincere characterization of them. Shrum (2017) attests that the media has the potential to transmit powerful narratives of repression as well as resistance through representations. Hence, there is an urgent need for an evolution in trans representations in Indian cinema in order to bring about progressive attitudes in the minds of the audience.

## 2 Review of literature

### 2.1 Lack of positive representations in Indian cinema

Transgender visibility in Indian cinema is a much-debated topic, particularly regarding how films represent gender diverse communities that resonate or dissonate with the dominant perceptions. In the context of Indian Cinema, films have invariably objectified and stigmatised trans identities thereby creating anxiety and fear of social aversion, hindering gender diverse people from coming out with their identified genders. These aspects are reflected through the lenses of Framing and Queer theories in Bollywood films like *Darmiyaan* (1997), *Tamanna* (1997), *Sangharsh* (1999), *Shabnummausi* (2005), *Welcome to come to Sajjanpur* (2008), *Murder 2* (2011), *B.A. Pass* (2012) and *Rajjo* (2013) which foreground transphobic notions reinforcing patriarchal hegemony (Yasin et al., 2020). For an extended period, trans sexualities have been considered a taboo and this marginalisation has been critically examined in bollywood and OTT representations through intersectionality and critical race theories, revealing that most of the depictions are stereotypical, inadequate and oversimplified (Qadiri, 2023). Similarly, Gogoi’s (2020) contrapuntal reading of the subjectivities of trans individuals unveils the stereotyped motifs, the rejection of trans community and the absence of a legitimate sex code for marriages between trans couples through Rituparno Ghosh’s *Chitrangada: The Crowning Wish* (2012). Also, Raveendran and Pannikot (2023), drawing from Butler’s theoretical framework of doing gender, identified how trans portrayals are often constrained to fit into a binary system in the Indian context through the films, *Chitrangada: The Crowning Wish* (2012) and *Super Deluxe* (2019). These cisheteronormative binary constraints on gender lead to varied modes of transphobic violence including trans-misogyny as observed by Nair and Francis (2024) in *Nanu Avanalla Avalu* (2015) and *Njan Marykutty* (2018). Moreover, a qualitative analysis quotes that there is no evident transformation in the cinematic depictions of transgender people in Indian cinema despite the legal recognition of transgender rights (Kaur and Singh, 2025). In addition, the impacts of binary gender norms, the historical contexts of gender segregation, and the commercial dynamics of queer film production in India are studied through a converging lens of queer, postcolonial and media studies by Rafiq et al. (2024).

### 2.2 Evolutionary and challenging transphobic representations

Moving away from the pattern of studying only pessimistic renderings of transgender members as deviant, impotent and villains in films, Afshana and Din (2017) identified less offensive depictions of transgender population in Indian films ranging from 1990 to 2010. They provided a systematic study by employing Butler’s Gender Performativity theory which posits that gender categories influence how individuals are expected to express their masculinity or femininity and create meanings out of them. However, trans characters in *Ardhanaari* (2016) and *Njan Marykutty* (2018) who perform their identified gender do not conform to any of these categories and exist in a liminal space embracing their abject status, which in turn challenges the gender oppression (Varghese and Tanupriya, 2023). Additionally, transfeminist framework has also been applied to conduct affirmative studies on the lives of trans communities, for instance, Kuriakose (2020) has used this framework in Malayalam films, namely, *Aalorukkam* (2018) and *Njan Marykutty* (2018) to strengthen their legitimacy and highlight their humanised characterisations. A systematic review of trans depictions in Malayalam movies from 1978 to 2024 has been done by Rahoof et al. (2025) from ecological perspective tracing the trajectory of LGBTQIA+ representation in Malayalam cinema. In the contemporary films, directors are experimenting with the scope and nuances of trans lives as in *Nagarkirtan* (2019) and *Chitrangada: The Crowning Wish* (2012) differing from their earlier insignificant and vile characterisations, as affirmed by Pillai and Sreenivasulu (2024) through Interactionist theory. Additionally, Vijayapadma’s *Narthagi* (2011), Seenu Ramasamy’s *Dharmadurai* (2016), Ram’s *Peranbu* (2018), Sudha Kongara’s *Thangam* (2020), Abhishek Kapoor’s *Chandigarh Kare Aashiqui* (2021) and Pa Ranjith’s *Natchathiram Nagargiradhu* (2022) are few of the other Indian films exemplifying the shift of perspectives on transgender. Chayani and Sahoo (2025) investigate trans representations in Indian cinema from the 1990s to 2023 through Foucauldian and Butlerian lens to trace the metamorphosis and find that contemporary Indian cinema has undergone a transformation in transgender portrayals but it has to go a long way to claim itself as “transgender friendly.”

### 2.3 Trans depictions in Tamil cinema

Tamil cinema has the second largest base in the Indian film industry and so, there is a need to enumerate the research that has been done on trans depictions in Tamil films. Besides Bollywood and other regional cinema, the Tamil film industry has been of great importance since its inception as the social, cultural and political ideologies of Tamil Nadu are incredibly influenced by films, unlike any other region in India (Damodaran and Gorringe, 2017). Hence, the portrayal of transgender population in Tamil cinema is significant due to the pressing need to sensitize people about the trans community in Tamil Nadu (Ravindar, 2018). It is identified that Tamil films have representations; however, it is not proportionate to the number of films being released every year and there is also a dearth of sincere representations of the trans communities that profoundly explore gender inclusivity. Further, the employment of cisnormativity as the master narrative framework in Tamil Cinema visual culture is questioned through the analysis of the film, *Super Deluxe* (2019) (SA, 2022). Mangayarkkarasi (2019) identifies transphobic depictions in the movies, *Eeramana Rojave* (1983), *Thullatha Manamum Thullum* (1999), *Appu* (2000), *Jayam* (2003), *Thiruda Thirudi* (2003), *Paruthiveeran* (2007) and *I* (2015) and emphasizes the need for proper awareness of trans identities through cinema to reduce prejudice and improve their opportunities for education, jobs and living standards in society. As Tamil cinema has the capability to critique the operations of the hierarchical gender system, resist heteronormativity and uphold inclusivity in the land (Varghese and Tanupriya, 2023), it can be used as an apparatus to influence the ideologies of the people in Tamil Nadu as the Tamil film industry “has grown to become the most domineering influence in the cultural and political life” (Baskaran, 1996, p. xii).

## 3 Research gap

### 3.1 Dearth of research in progressive trans depictions in Tamil cinema

The hither-to research has dealt with negative portrayals and the evolution of trans communities in Indian Cinema, especially Tamil cinema. The current research concentrates on the potential of trans narratives in Tamil cinema as a medium of communicating positive and authentic lived experiences. The present research throws light on the potential of the progressive portrayal of transwomen in Tamil Cinema.

### 3.2 Lack of research on interaction depictions between cis and trans in tamil cinema

Previous research focused on the misrepresentation of trans characters in isolation. The current study delineates on the nature of depicted intergroup interactions between the trans and cis people in the select Tamil films.

### 3.3 Negligible research on the select movies from transgender lens

In this research, the select films, *Peranbu* (2018) and *Natchathiram Nagargiradhu* (2022) are viewed through transgender theoretical perspective, unlike the previous research exploring the themes of disability (Francia et al., 2024) and casteism (Bunkar, 2024) respectively.

## 4 Objective

The current study intends to explore the extent of accurate and positive portrayal of trans identities in Tamil cinema by analysing the positive interaction depictions between cis and trans members in select films, *Peranbu* (2018) and *Natchathiram Nagargiradhu* (2022), through a combined framework of Integrated Mediated Intergroup Contact model and Trans-Identity theory.

## 5 Theoretical framework

To investigate the interaction depictions and characterisations of trans members in select films, the paper proposes a linkage of dual frameworks—the Integrated Mediated Intergroup Contact (IMIC) model (Wong et al., 2022) from media studies and Trans-Identity theory (Nagoshi et al., 2014) from Gender studies to ascertain whether positively mediated intergroup contact between cis and trans groups leads to affirmation of trans identities aligning their lived experiences. The IMIC model has been instrumental in integrating both parasocial and vicarious forms of mediated contact to reduce prejudice. Mediated parasocial contact occurs when viewers establish a connection with the marginalised or outgroup member of society when portrayed in the media. It elicits positive parasocial responses wherein a media consumer interacts and creates one sided relationship with an outgroup character (Schiappa et al., 2005), allowing them to reevaluate their biases and develop empathy. Conversely, mediated vicarious contact gives positive parasocial responses through narrative transportation, where the viewers are actively observing the intergroup interactions. During the interactions, the viewers identify with ingroup characters or figures representing the dominant group, enabling modification in their behaviors towards outgroups with an increase in self efficacy and decrease in intergroup anxiety. According to Wong et al.’s (2022) IMIC model, both mediated parasocial and vicarious contact lead to prejudice reduction, provided that the nature of contact is positive. Hence, the positive mediators responsible for deriving prejudice reduction given by Wong et al. (2022) are considered to study the mediated intergroup contacts in select films.

Furthermore, the lived experiences of the outgroup characters depicted through mediated intergroup contact should be assessed positive or negative. The study considers trans people as outgroup or minority members and cis people as ingroup or dominant members. To decipher the nature of mediated intergroup interactions from transgender (outgroup) perspective, Trans-Identity theory (Nagoshi et al., 2014) is employed. Trans-Identity theory integrates physically embodied experiences with the self constructed and socially constructed aspects of identity, informed by the lived experiences of those with fluid, intersecting identities (Nagoshi et al., 2023).

The first aspect of Trans-Identity indicates that, aligning with feminist scholarship and Shotwell and Sangrey’s (2009) theorization, there is an explicit correlation between the identity of a trans person and their physical embodiment; referring to the Self, which is generated from bodily experiences (Nagoshi et al., 2014). The second aspect coincides with the feminist and the queer theorists in maintaining that the process of affirming the self constructed identity originates from the subversion of socially imposed identities and emanates from the narratives of their lived experiences (Nagoshi et al., 2014). The refinement introduced by Nagoshi et al. (2014) is the third aspect, which is the socially constructed identity born out of “repeated performances” of the societal gender roles associated with the identified gender, resulting in an “objective identity” in an effort to belong to the identified gender. They posit that an “autonomous self” emerges only when there is an integration and interaction among the physical embodiment, the rebellious self constructed identity and the conforming socially constructed identity aspects. Thus, trans identities necessitate an analysis of their lived experiences through these three interconnected aspects.

The paper attempts to assess the intergroup encounters between cis and trans members in the select films *Peranbu* (2018) and *Natchathiram Nagargiradhu* (2022). The potential of trans women representations in the selected films focus on situating these intergroup situations within the nexus of embodied experiences, their self-constructed and socially constructed dimensions of identities.

## 6 Methodology

### 6.1 Materials

The study explores two Tamil films, namely, Ram’s *Peranbu* (2018) and Pa Ranjith’s *Natchathiram Nagargiradhu* (2022) to investigate the depictions of positively mediated intergroup contact between cis and trans communities and the optimistic manifestation of transgender identities in them. *Peranbu* (2018) and *Natchathiram Nagargiradhu* (2022) received worldwide acclaim and bagged the praise of many eminent film directors and critics across India. The rationale behind choosing these contemporary films as primary sources is that they feature real trans women actors in a positive role which is new to the Tamil audience and also depict dignified intergroup interactions between cis (ingroup) and trans (outgroup) communities. The directors of the select films affirmed in movie promotions that they have crafted the trans characters after interacting with real trans women and hence intend to break the stereotypes by portraying them with sensitivity and sensibility, unlike earlier portrayals. The data used here includes select scenes, characters, images, metaphors, dialogues and mediated contexts that the researcher finds relevant to the objective.

### 6.2 Methods

The paper considers the select films *Peranbu* (2018) and *Natchathiram Nagargiradhu* (2022) as visual texts and employs a qualitative approach for the scrutiny of the same. Textual analysis has been adopted by the researcher to ascertain the progressive portrayal of trans women in the select films. Firstly, to familiarise with the text, the films have been watched multiple times to uncover the explicit and implicit messages delivered on screen. The study focuses on specific scenes, images, characterisations, subtitled dialogues, intergroup contexts involving trans communities and the interaction depictions between cis and trans communities. The researcher attempts to interpret the frames and actions through close reading of the cast, the background setting, the dialogues, the character development of cis and trans characters, the dignity associated with the trans characters, the relevant context connotated and the instances where transphobia and transrespect are exhibited. The analysed content and its interpretations are systematically grouped under the four themes adopted from Wong et al.’s (2022) Integrated Mediated Intergroup Contact (IMIC) model, listing the positive mediators of mediated intergroup contact, both parasocial and vicarious forms. The sections compare and contrast the selected scenes with the transphobic depictions in earlier films to bring out the instances of positive intergroup interaction located in the selected films. Further, the analysis of the identified positive mediators in the films is verified for progressiveness by closely reading them against the three aspects of Trans-Identity Theory (Nagoshi et al., 2014) to comprehend the authenticity of the trans experiences portrayed, to achieve the ultimate outcome of the intergroup contact, i.e., prejudice reduction towards trans people.

## 7 Mediators of Integrated Mediated Intergroup Contact in *Peranbu* and *Natchathiram Nagargiradhu*

Positive and accurate media representations of trans people and interaction depictions between cis and trans groups are important to remove the misconceptions carried by cis people, perpetuated through stigma and stereotypes and to assure trans people that their experiences are acknowledged and shared by others as well (Orellana et al., 2022). Research conducted on positively mediated such intergroup interactions exclusively concentrates on either parasocial (encounters a marginalised character through media) or vicarious (observing dominant character’s interaction with marginalised character) forms of contact. Ray and Husnu (2025), emphasize on parasocial contact to improve positive outgroup attitudes and garner support for transgender rights through perspective taking. Alternatively, Mazziotta et al. (2011) suggest that when the representations are done through mediated vicarious intergroup contact, it elevated the viewer’s self efficacy by reducing the anxiety and improved the attitudes towards minorities or outgroups. Further, in vicarious contact episodes, outgroup narrative perspectives yield more positive attitude changes than those from dominant or ingroup perspectives (Li, 2019). The current study locates the scenes where mediated intergroup contact between trans and cis identities happen in *Peranbu* (2018) and *Natchathiram Nagargiradhu* (2022) through both parasocial and vicarious forms employing the Integrated mediated intergroup contact model and the trans positive characterisations in these interactions are delineated with Trans-Identity Theory.

### 7.1 Positive parasocial contact

#### 7.1.1 Positive parasocial response as a mediator

Parasocial relationships are an alternative to relationships formed socially through direct contact, where the viewers relate themselves with the mediated characters (Cohen, 2009). When facilitated through media, parasocial contact has the capacity to foster prejudice reduction (Schiappa et al., 2005). The responses derived from parasocial contact comprise empathy and attachment that a viewer constructs with the mediated figure (Bond, 2020). The empirical relationship between the mediated exposure of the marginalised communities and the parasocial responses of the audience was analysed by Banas et al. (2020), revealing that positive mediated parasocial contact occurrences significantly mitigate prejudice against outcast groups. These results are extended to the entertainment media contexts by Wong et al. (2022), which are applied to the films under consideration in this paper.

To develop an enriching parasocial relationship with the outgroup characters, particularly trans characters in films, they should be authentic and evolve into likeable characters over the course of the plot (Schiappa et al., 2005). On the contrary, bleak and unfavourable representations of gender non-conforming personas without positive character development have been the content in many popular movies, including Vasanth’s *Appu* (2000), Gautham Vasudev Menon’s *Vettaiyaadu Vilaiyaadu* (2006) and Shankar’s *I* (2015). Moreover, directors cast cis men in roles meant for trans women (Ulaby, 2014), due to the social interpretation that trans women are actually male who then turn out to be imitating feminine behavior (Nagoshi et al., 2023). Due to this insensibility practised, the characters often lack believability and appear clichéd and positive parasocial responses are not formed. Films that had been well received by the audiences for their trans portrayals, such as Raghava Lawrence’s *Kanchana* (2011), Thiagarajan Kumararaja’s *Super Deluxe* (2019), and Sudha Kongara’s *Thangam* (2020), are also misrepresentations as they reduce the complexities of trans experiences to mere cross-dressing by male actors. Those who wear attire associated with the opposite gender are typically considered cross-dressers (Ravindar, 2018). Due to these negative and reductive portrayals, the parasocial interaction leads the audience to misconception about trans identities. But the trans women characters in *Peranbu* (2018) and *Natchathiram Nagargiradhu* (2022) are played by real trans women Anjali Ameer as Meera and Sherin Celin Mathew as Sylvia which will add credibility to trans characterisations and avoid misconceptions.

The plots of select films also give due importance to the positive character development of the trans cast enhancing their on-screen presence, likeability and dignity leading to positive parasocial connections with the outgroup characters. Their strong dialogues and subtle expressions have far-reaching effects in breaking the stigma around them and showing their essential roles within society. Meera, in *Peranbu,* exemplifies compassion, serving as a pivotal figure in the life of the protagonist Amudhavan. She comes as a compassionate saviour in his life as she helps him in all possible ways, from finding a house to taking care of his spastic daughter, Paapa. Her characterization reaches the pinnacle when she intervenes and saves Amudhavan and Paapa from their tragic fate in the climax. Amudhavan, intending to commit suicide, drags Paapa along with him to drown in the ocean due to the overwhelming despair of living in an unkind world. When Meera sees them, she rushes to rescue and drags them to the shore. As her name “Meera” connotes the ocean, she enters their lives with her lifegiving spirit of the ocean and coalesces with them to form a family and, with her abundance of love (Peranbu), creates a home which is beyond the worldly conventions of a heteronormative family.

Similarly, another trans outgroup character, Sylvia, in *Natchathiram Nagargiradhu*, stands for the upright qualities: resilience and political correctness. Her characterisation is marked by rationality, which is evident when she voices her valid points on the politics of gender and casteism in love marriages when the theatre crew discusses staging a play on the politics of love. The truth is conveyed boldly by her and it is credible when the protest against the societal dogmas comes from her, as she is a resilient being living in a sabotaging patriarchal society. Sylvia is portrayed as an intellectual rationalist and the discussion below can be an instance of her rigour against discrimination.

Subeer: Love is evolving across the world. But here, especially in Tamil Nadu, honor killings are in the rise. Even now…

Arjun: Master! Not all love stories face this fate. Does it? Only the “show off fakes” face this.

Karpagam: According to you, clothes and bikes are all women need to fall in love, right? Let me speak. There isn’t a worse insult.

Arjun: I didn’t mean that, okay. But that is the truth. I have seen so many people. They possess nothing absolutely. But they dress up so perfectly.

Sylvia: Their attire isn’t your problem. You want those “nobodies” to remain as they are. That is the problem. Your daughter chooses this “nobody” and you get all worked up (Ranjith 2022, 00:30:00).

When these parasocial relationships are formed, the audience are involved with the outgroup characters on cognitive (perspective taking), affective (empathy) and behavioral (interacting with the character) levels (Schramm and Hartmann, 2008; Wong et al., 2022). As a result of having a likeable role and positive character development, when trans women characters speak of their anxieties and afflictions, the audience seek to comprehend the thoughts of the character and empathize with them, consequently, understanding the troubles of their community (Schiappa, 2024). When an injustice is done to them, people react accordingly, in lieu of apathy derived from earlier depictions. Thus, trans characters grow up to be viewers’ favourites and the representations informing the truth allow viewers to form one-sided relationships with them whose realities are hardly accessible. Through parasocial bonding, the traits that are attributed to the particular categories will be altered if the experience has been productive enough to facilitate changes in beliefs and attitudes toward the particular social group (Schiappa, 2024).

#### 7.1.2 Identification with outgroup character as a mediator

As per previous studies, in mediated parasocial interactions, the viewers often identify themselves with marginalized or outgroup characters. This process of identifying with the ostracized members on screen increases the empathy towards the oppressed individuals in the real world and have an overall shift in the perspectives towards the represented outgroup (Wong et al., 2022). According to Cohen (2013), identification happens “based on a series of momentary connections with a fictional character or performer. Thus, any insights, memories, feelings, or knowledge that (one) experiences through (one’s) identification with media characters is integrated into (one’s) life.” Moreover, the impact on the audience is not affected by the duration of the exposure but on the dignified presentation of outgroup characters (Banas et al., 2020). Both positive and negative reinforcement happens regardless of the minimum or maximum time of exposure.

Earlier, negative depictions of trans women in Tamil films, for instance, *Paruthiveeran* (2007) and *Sillunu Oru Kaadhal* (2006), have often been employed to arouse laughter by ridiculing their bodily differences, and were relegated to brief appearances, majorly for comic relief (Veena, 2024) by portraying them as “stupid, silly, lazy, irrational, or simply laughable” (Fitzgerald, 2010). They entail skewed perceptions towards trans people, furnishing undesirable roles of “sex-workers, mentally ill, freaks, self-mutilators, cripples, criminals, and as unlovable” (Davis, 2009). Trans women had been cast in movies exclusively to be insulted and shamed based on their gender expressions, provoking the base instincts of the throng to derive pleasure by inflicting hurt on these communities. These stigmatizing movies show the prejudice over trans people and are not suitable for positive parasocial contact. In fact, such negatively mediated contact more strongly reinforces negative stereotypes than positively mediated contact reinforces positive attitudes (Banas et al., 2020) and finding “positive trans role models are rare” (Davis, 2009).

Over the years, trans people have been portrayed as ‘the other’ in the heteronormative discourses” of Indian cinema and were often seen “as victims, social outcasts, and diseased or disordered” (Nair and Francis, 2024). In *Peranbu,* the vulnerable status of a trans woman is evident when Amudhavan happens to hear a person wailing for help in the outskirts; he runs to the rescue and encounters Meera, a trans woman being physically assaulted by a whoremonger in an abandoned place. He saves her, gives her water and offers to take her to the hospital but she refuses to take the help; she thanks him and leaves breaking the stereotypical portrayal of trans members as beneficiaries and cis members as benevolent members of the society. She does not expect his mercy and is not reduced to being a victim without agency seeking help from an ingroup cis character. She extracts empathy from the audience, similar to the ingroup character empathizing with her. She urges the audience to react to the injustices meted out to trans women by telling her liability to danger and threat in society where the viewers live without incessant fear.

Amudhavan: (Rescuing Meera) Here, drink some water. Oh no, you are bleeding? Who’s that guy?

Meera: More than half our customers treat us like this, like beasts.

Amudhavan: Shall we go to the doctor? I’ll take you.

Meera: It’s okay sir. My people will be standing there. I’ll go. Thanks, sir (Ram, 2018, 01:41:34).

The outgroup trans character, Sylvia, in *Natchathiram Nagargiradhu* speaks for her community which is pushed to the margins, bringing out their concerns and their difficult romantic relationships. The crew also acknowledges the rationale with which she tells without subduing them as tabooed topics. In all the discussions held, Sylvia’s thoughts and words are not dismissed but held with great value surpassing the stereotype of casting them as incompetent and irrational. These depictions bring out her experiences of love, lust, anger, agony and resilience which can make space for cognitive, emotional and behavioral responses from the viewers.

Sylvia: Joel and I are witness ourselves. So many transgender people like me are still struggling. Somehow, we can live together. Only love can shatter gender barriers (Ranjith, 2022, 00:28:36).

In order to acquire positive intergroup connections with good insights, memories, feelings or knowledge associated with the depicted outgroup (trans) character, the mediation in the films should integrate the embodied experiences and its social aspects as seen in the select films. The intersecting nature of their experiences is crucial to be considered when crafting a complex outgroup character, which by extension, benefits the whole outgroup community (Wong et al., 2022).

##### 7.1.2.1 Empathy

Positive parasocial response and identification with outgroup character give rise to empathy (Wong et al., 2022). The characterization of Meera in *Peranbu,* culminates with the metaphorical image of the lighthouse on the seashore. She becomes a beacon of hope in the lives of Paapa and Amudhavan on their dark days, illustrating her capacity to spread love despite her trauma of societal rejection. When the outgroup character is depicted in a positive light, viewers connect and identify with the outgroup character and positive parasocial responses are created, consequently, increasing empathy. Anjali Ameer, the trans woman who played the character Meera, admits in an interview that people in her hometown see and treat her with respect after watching her portrayal in the movie. She informs the interviewer,

“I received a lot of congratulatory messages. But what was more uplifting was that many who had previously shown antipathy towards me as a trans person expressed a change of heart. They acknowledged that they could see the transgender community in a new light through Meera” (Harikumar, 2019).

Similarly, Sylvia in *Natchathiram Nagargiradhu* is shown as a convivial member of the theatre crew and has politically sound ideologies on gender, caste and religion. The audience are awestruck with the clarity she speaks on casteless, genderless love. The viewers empathise with her intelligence being belittled by the patriarchs like Big Cat due to her transgender status, in the film.

“The film’s queer and trans characters are three-dimensional, a refreshing break from mainstream caricatures. Their love and struggle for identity is shown like any other” (Bag, 2023).

These statements stand as testimonies of the resulting empathetic responses towards the mediated characters which broke the stereotypical representation.

### 7.2 Positive vicarious contact

#### 7.2.1 Narrative transportation as a mediator

Positively mediated vicarious contact occurs by observing positive interactions between ingroup and outgroup members (Ortiz and Harwood, 2007). In vicarious contact, parasocial response and identification with outgroup and ingroup characters happen through narrative transportation (Wong et al., 2022). Narrative transportation, characterised by the immersion of viewers within the storyline, heightens identification with the characters, fostering a state where they absorb ideologies with minimal resistance according to Slater and Rouner’s (2002) extended elaboration likelihood model. This transportation entails a desirable attitude formation of viewers towards represented social minorities (Wong et al., 2022). In a positively mediated vicarious contact, the movie characters have to be portrayed authentically in order to establish powerful connections between the movie characters and the viewers who are being transported into the cinematic world. Spectators, when being transported, are persuaded to believe the messages being conducted on screen and hardly resist them as they identify themselves with the mediated figures.

There are ample instances of cis gender (ingroup) and trans gender (outgroup) members interacting with each other in media where “cisnormative expectations and norms pose obstacles to open conversational exchanges” (Heinz, 2018). Differing from the earlier intergroup environments created in films, Pa Ranjith in *Natchathiram Nagargiradhu* has created a non homogenised universe where everyone has their individuality and can exhibit their uniqueness without the fear of being isolated. The Indianostrum Theatre and its ensemble emerge as a prototype for the non heteronormative world, effectively transporting the audience into a space constructed by theatrical creativity and inclusivity beyond race, caste, gender and sexual orientation.

The diverse ecosystem of the theatre group that holds space for everyone, dismissing the conservative ideals of the society is resonated through the lyrics of the song,

“Beat the drum, utter it loud

The world is equal for all, say it!

No matter where you are from

or who you are” (Ranjith, 2022, 00:20:10)

The narrative universe gives a chance for the viewers to come in contact with a utopian space which is nearly impossible in the real world. In this microcosm, discomfort arises only by being conservative as illustrated by the ingroup character Arjun. When Sylvia, a trans woman character warmly extends her hand introducing herself, he hesitantly shakes hands with her, displaying the anxiety of coming into direct contact with a trans woman. It also underscores the pervasive stigma and discrimination trans people face in intergroup environments. He then further goes on to namecalling her to Shekar. But, Shekar, unlike earlier dominant cis characters in films, instead of joining Arjun in derisive laughter reprimands him for his vulgarity and teaches the right way to address her, modelling a respectful approach.

Shekar: One slap! Where did you study? What the fuck did you learn? Come close. Trans woman. Alright? What is the term?

Arjun: Trans woman.

Shekar: That’s right (Ranjith, 2022, 00:17:54).

The scene is crafted by intentionally obscuring Arjun’s derogatory term and ensuring that the proper terminology to address her is being articulated explicitly and emphatically by Shekar contrasting with previous cinematic storylines. The director uses the mediated vicarious contact to show how, “applying proper terms to refer to trans people in media, increasingly legitimizes trans individuals; in contrast to name-calling them and using incorrect pronouns, which delegitimizes them” as emphasized by Van Haelter et al. (2022). The film focuses on building an intergroup environment with the consciousness of being inclusive of everyone, that audience can model upon while constructing their intergroup communication.

Likewise, in *Peranbu,* there is an incident where Amudhavan rescues Meera from harassment and offers to help her, but she declines help and leaves the place thanking him. A fellow cab driver who has been witnessing everything from the cry for help till that moment, remains unaffected by the injustice and only warns Amudhavan of the harmful consequences of getting involved and helping the trans people due to the stigmatization surrounding them.

Another Cab driver: Sir…those things are neither male nor female. If you get into their problems, you’ll be screwed (Ram, 2018, 01:41:34).

Here, the cab driver reduces the identity of Meera to that of a creature but Amudhavan stands still and could neither retaliate nor accept what he uttered about her because he did not have the awareness of her lived experiences. And in another scene which unfolds below,

Meera: Do not feel shy. Tell me what it is? You still have not said what you wanted to say.

Amudhavan: Do you know any brothels nearby?

Meera: What?

Amudhavan: No… You know these kind of…any brothels around here?

Meera: I maybe someone who does business on the road for cheap money. But I don’t know these kind of places (Ram, 2018, 02:10:55)

Here, Meera herself retaliates and teaches Amudhavan to interact appropriately with her. The film gave trans women the opportunity to negotiate or retaliate on the stereotypical traits burdened on them causing societal misunderstandings. The usual trope of showing a trans woman as a helpless sex worker, self stigmatizing herself and submitting to insults is renegotiated in the film where Amudhavan realizes his limited perspectives on a trans woman as he peers deeper into her life in this transphobic world. It should be noted that *Peranbu* makes an effort to drive home the intended messages from trans perspectives with the awareness that the audience will be influenced by the persuasive nature of the film.

The exchange between the ingroup and outgroup characters in the select films illustrates the necessity of behavioral education towards outgroups and an inclusive language to be used by ingroup characters in films while addressing outgroups, as the audience highly tend to absorb and recreate the depicted intergroup behaviors.

#### 7.2.2 Identification with ingroup character as a mediator

Observation of intergroup interactions between the dominant ingroup and the marginalised outgroup characters in media is facilitated through mediated vicarious contact without having the anxiety of being directly involved in it. The intergroup anxiety associated with directly interacting with the outgroups is diminished, thereby enhancing the propensity to form bonds with the other social groups (Moyer-Gusé et al., 2019). The viewers would identify themselves with the social groups that correspond with their memberships which can be referred to as the ingroup in intergroup interactions. Drawing from the evidence of the IMIC model of Wong et al. (2022), it is understood that when individuals within a particular group are depicted to be interacting with the opposite groups in a dignified manner on screen, it steers agreeable attitudes towards the outgroups.

But film creators have often depicted transphobic interactions; trans people were either made a laughing stock for furnishing poor humour or shown as monsters to create fright. For instance, in Shankar’s movie *I*, Osma Jasmine, a trans lady is portrayed as an abhorrent and immoral individual just for the fact that she belongs to trans outgroup, even though she is in the position of the sought-after stylists. She is teased because she is seen as a male behaving effeminate, thus invalidating her identified gender as a woman. The characterisation is set to produce terrible repulsion influencing the point of view of the spectators. Popular trans people namely Rose Venkatesan, a TV anchor and Living Smile Vidya, author of India’s first trans autobiography and an eminent theatre artist, called out *I* (2015) for its callous depictions and the typecasting of trans characters in negative shades realising the power of films to influence the audience. The creators regulated the heteronormative perspectives by enabling identification with flawed ingroup characters, transportation into a patriarchal world and producing transphobic responses (Moyer-Gusé, 2008), which in turn contributes to ‘real-world transphobia’ (Flew, 2021).

Offering a stark contrast and employing the cinematic device of a play with a play, Ranjith, the director of *Natchathiram Nagargiradhu* gives an instance where flawed ingroup characters are employed to dysregulate heteronormative perspectives. Embodying an upper-caste patriarchal perspective, the ingroup character Arjun passes derogatory comments and indulges in insensitive actions in the film. He blatantly upholds the dichotomies between the binary and non binary gender dictated by his upbringing. He degrades the love shown by outgroup characters, people of non binary gender, and cannot bear the sight of a trans woman or homosexual couples in the theatre. He reflects the deeply entrenched societal biases, but unlike in the real world, he faces derision for his regressive beliefs in the film. The flawed character, Arjun sits down to watch the drama rehearsals and he finds himself terribly uncomfortable with the behaviors of the ingroup characters in the play. As the tension rises towards the climax of the play, he senses his ideological flaws and understands that it reflects his mob mentality. Arjun’s sensitization and behavior modification through identification with ingroup character in the play can be compared to the nature of influence the movie would have on the viewers. The excerpt below can be likened to the reforming experience of the audience as well which can be seen below:

Arjun: How conservative I was when I came here. After coming here and meeting you all… a lot has changed in me… Just like my change of heart, my parents would change too and I believe that. So, I think this would be a positive approach.

Also, in *Peranbu*, the audience identify with the ingroup character, Amudhavan, and relate with the metamorphosis of his perspectives on Meera, from seeing her as a prostitute, a passive objectified transgender character, to Meera as a compassionate human being actively resisting transstigma during the course of the movie.

Amudhavan’s realization can be witnessed when he says,

Amudhavan: I had to plunge into an ocean and emerge out of it…to discover something there is called compassion. And the person who made me discover this, is my wife, Meera (Ram, 2018, 02:21:48).

The film calls upon the audience to witness the world of Amudhavan, Meera and Paapa and comprehend their ways of lives which fights against the system which deems transwomen undeserving of filial love as her body is devoid of womb. In the final chapter entitled “Nature is compassionate,” Ram showcases an unconventional happily ever after where the trans woman Meera and the disabled child Paapa are seen to be coexisting harmoniously as a family with a cis male Amudhavan.

Amudhavan exclaims, “Thus, I was fathomed that love and affection are beyond such differences” (Ram, 2018, 00:42:54) in the hope that the audience would fathom the same.

##### 7.2.2.1 Self efficacy

Self efficacy can be defined as “the self-perception that an individual has the ability to enact a behavior” overcoming their biases (Krcmar, 2019). The very “knowledge that an ingroup member has a close relationship with an out-group member can lead to more positive intergroup attitudes” causing “anxiety reduction” by “reducing ignorance” (Li, 2019; Wright et al., 1997, p. 73). The mediated contact process extends beyond mere identification with characters to be materialised into action, for the reinforcement to be done in the real world. To call for an action, the media should portray that the members who initiated intergroup interaction overcoming their anxiety and social conditioning are rewarded for their positive behavior so that the self efficacy of the viewers also increases. Both the ingroup and outgroup members should gain self efficacy to promote communication between them (Wong et al., 2022).

The transgender groups have historically faced systemic social exclusion within a discriminatory milieu. The phenomenon of social isolation, both real and perceived, manifests in interactions that infringe upon personal boundaries, reducing identities solely to their transgender status, thereby reducing self efficacy (Heinz, 2018). On the contrary, the act of Amudhavan interacting and treating Meera with respect and dignity disrupts the social pattern of isolating trans women and focuses on her intrinsic humanity restoring the self efficacy of Meera. When Meera comes out with her acts of love and Amudhavan takes the initiative to transcend all the norms of society and begin their journey as a family which reflects the self efficacy of Amudhavan in abstaining from prejudice and embracing humanity.

Similarly, Joel and Sylvia are shown as a couple in *Natchathiram Nagargiradhu.* Joel and Sylvia practice self efficacy as they are surrounded by an inclusive community with progressive ideals. Sylvia admits in a discussion that Joel “is the only one who believes that I am a woman. So, I decided to love him” (Ranjith, 2022, 00:48:08). The film shows the transformative role that social acceptance has on self efficacy. Arjun also is a suitable instance for how self efficacy to go beyond social conditioning increases when a politically aware and supportive social environment is available. Thus, Amudhavan, Meera, Arjun, Joyel and Sylvia efficiently transcend intergroup anxiety when self efficacy increases and when the anxiety to interact diminishes and they enjoy their bounties.

### 7.3 Integration of physical embodiment, self constructed identity and socially constructed identity

The select films attempt to document the lived experiences of trans identities in a social context during intergroup contact scenarios from the lens of Trans-Identity Theory. The theory emphasizes that in order to comprehend the lived experiences of trans people, their physically embodied, self constructed and socially constructed identities are to be taken into consideration. The exemplar themes which are considered as the mediators of intergroup contact in each of the above sections are analysed if they are authentically showcasing trans identities. In parasocial contact, trans community members are cast for desirable trans roles in the select movies which gives credibility to the depicted trans narratives. The act of inclusion of trans women Anjali Ameer and Sherin Celin Mathew, recognizes their self identified genders and physically embodied aspects of identities. Hence, the films are progressive in the way that they are sensitive to the fact that casting cis male actors instead of trans women actors is an act of invalidation of their physical embodiment and self constructed identities as trans women feel inauthentic to embody their assigned gender at birth after they have transitioned into identified gender.

The trans characters, Meera and Sylvia do not neglect or conceal their trans identities at any point of time in the films. They physically embody femininity, resistance, pain, beauty and endurance and their self constructed identities are born out of subverting socially imposed labels on them. The characters strive to break the stereotypes of being the victims and criminals in the earlier films by bringing out the hardships of their communities and putting it out for discussion so that the audience can identify with their struggles. They are portrayed to be self affirming of their gender and refuse to lower their dignities at any point of time. Self assertion of trans identities is of prime importance in the film as it helps the viewers cultivate an understanding of transgender and change their outlook on outgroup community as a whole. The legitimisation is strengthened with the positive character development, validation and acknowledgement gained through positve parasocial responses and identification with outgroup characters effecting empathy. Hence, care should be taken to depict trans people in an authentic manner due to their potential impact on the viewers. Thus, positively mediated parasocial contact helps people to develop empathy towards trans outgroups though the public can hardly have direct contact with them (Wong et al., 2022) and understand their lived experiences.

**Figure 1:**
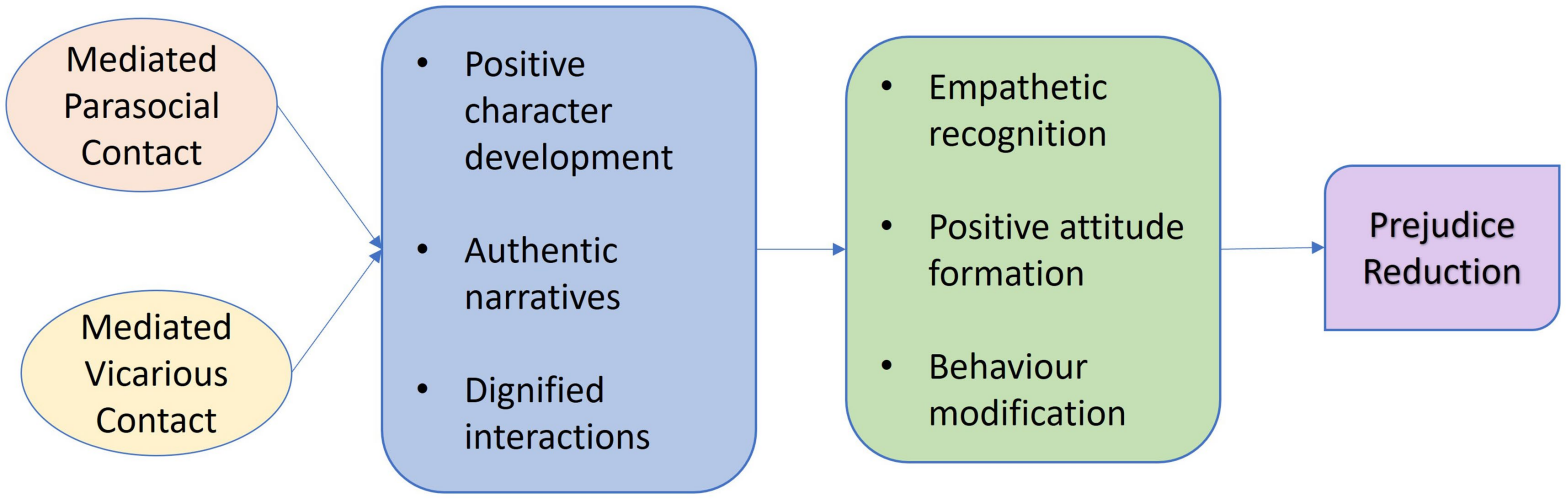
Model of trans prejudice reduction.

Analysing through mediated vicarious contact, the gender constructs in the earlier films were identified to be patriarchally dominant, that the depictions were too brutal to the self and socially constructed aspects of trans identities. In Ram’s *Peranbu*, and Pa Ranjith’s *Natchathiram Nagargiradhu*, the perspectives on and of transgender individuals are sincerely articulated in the intergroup interactions with an intent of eliciting behavioral responses through exemplifying appropriate intergroup behavior on screen as self identification and social acknowledgement of one’s gender is crucial for confirming one’s identity. In the select films, trans women assert their self identified genders by conforming to the societal meanings of their identified gender. According to Cooley’s theory of Looking Glass Self, external validation of the identified gender by the society has an indispensable role to play in trans-becoming (Pillai and Sreenivasulu, 2024). Meera identifies as a woman and to assert her self constructed identity, she socially conforms to the roles of a woman in Indian society by embodying the stereotypical feminine roles of providing care and love to Amudhavan and Paapa. In *Natchathiram Nagargiradhu*, Sylvia substantiates it by claiming that she is in love with Joel as he sees her as a complete woman. Though the social atmosphere where Sylvia or Meera lives in, people recognize and respect their self constructed genders, they are also noticed to be fulfilling conventional social norms of being a woman like being a mother, being in a relationship with male and providing for the family and so on. Additionally, “trans people who do not meet social expectations regarding a gender-congruent appearance are more likely to suffer discrimination” (Orellana et al., 2022; Platt and Milam, 2018). Catering to these expectations, the trans women characters have long hair, adorn themselves and conform to the societal dressing behaviors of their identified gender, while actively resisting to conform to their medically assigned genders. These nuanced representations show how conformity becomes a resistance in a trans woman’s life because embodying self constructed femininity and socially constructed familial or romantic relationships without a womb in her physiology is a mockery of the patriarchal family setup. Thus, there is an intermingling of physically embodied, self constructed and socially constructed aspects of identities in the trans representations of these movies which is essential for understanding the trans realities. These intergroup representations corresponding with the three aspects of trans identities result in prejudice reduction in the select films as represented in Figure 1.

## 8 Conclusion

The mediated exposure of transgender people in a positive light is crucial because there is a need for the identities of social minorities in the society to be acknowledged and accepted. The select films tell their “transcendent stories” (Ekins and King, 2006) where trans identities are generated from their experiential realities connected to their self, body and society. It is evident from the analysis that when the subjectivities and evolutions of trans characters are captured through authentic and credible narratives employing real trans actors, the audience would have enough trans role models to follow and establish parasocial relationships with them through media (Schiappa, 2024). Such depictions can elicit positive parasocial responses as they emerge from going beyond the stereotypes. The study also foregrounds that these “transgressing” (Nagoshi et al., 2014) narratives of lived experiences have a strong impact on the audience due to the persuasiveness of narrative transportation, as humans “live in a world erected by the stories they tell” (Gerbner, 1998). The mediated content depicting dignified interactions between cis and trans members allows the viewers to identify with ingroup and outgroup characters, paving the way to unlearn the negative preconceived notions caused by the heteronormative patriarchal narratives about trans outgroups. This study, similar to the quantitative study of Banas et al. (2020) on creating persuasive intergroup connections through media, observes that the select films mediating positive intergroup contact can be used to inculcate knowledge on trans experiences and persuade to modify their attitudes and behaviors towards trans people. Due to increased empathy and self efficacy, the viewers attempt to comprehend the lived experiences of trans people and subsequently, prejudice over trans people may be reduced.

## 9 Limitations of the study

First of all, the study employs a qualitative textual analysis of the select Tamil films and has not conducted any audience perception analysis to derive statistical results. Secondly, it concentrates only on two Tamil films which may not be exhaustive of all the experiences of trans women and the researcher lacks first hand experiences of the situations faced by trans people, solely depending upon the academic literature for reviewing and analysing their lived experiences.

## Data Availability

The original contributions presented in the study are included in the article/supplementary material, further inquiries can be directed to the corresponding author.
